# Outcomes of anatomic and reverse total shoulder arthroplasty in patients over the age of 70: a systematic review

**DOI:** 10.1016/j.xrrt.2023.02.003

**Published:** 2023-03-04

**Authors:** Favian Su, Prashant Nuthalapati, Brian T. Feeley, Drew A. Lansdown

**Affiliations:** a Department of Orthopaedic Surgery, University of California, San Francisco, San Francisco, CA, USA; b University of Texas at Dallas, Richardson, TX, USA

**Keywords:** Total shoulder arthroplasty, Reverse total shoulder arthroplasty, Elderly patients, Aging population, Functional outcomes, Complications

## Abstract

**Background:**

Both anatomic total shoulder arthroplasty (ATSA) and reverse total shoulder arthroplasty (RTSA) reliably improve pain and function for a variety of indications. However, there remain concerns about these procedures among elderly patients due to their general health, the potential for lesser functional gain, and the possible need for revision at an even older age. The purpose of this review is to compare the clinical outcomes, radiographic outcomes, and complications of ATSA and RTSA among patients older than 70 years.

**Methods:**

A systematic review was performed using searches of PubMed, Embase, and Cochrane databases. The inclusion criteria were studies with patients older than 70 years who were treated with a primary ATSA or RTSA and clinical results reported at a minimum of 2 years. All indications for primary RTSA except for tumor were included. Outcomes of interest included patient-reported outcomes (PROs), range of motion, patient satisfaction, radiographic changes, complication and revision rates, and implant survival.

**Results:**

A total of 24 studies met the inclusion criteria. At a mean follow-up of 3.4 years for ATSA and 3.1 years for RTSA, there were significant improvements in pain, range of motion, and PROs for both prostheses. Patients who underwent ATSA generally had better motion and functional outcomes compared to those who underwent RTSA, though these comparisons were made across different indications for arthroplasty. The satisfaction rate was 90.9% after ATSA and 90.8% after RTSA. Furthermore, 10.2% of ATSA patients and 9.9% of RTSA patients experienced a surgical complication, whereas 2.3% of ATSA and 2.2% of RTSA patients underwent a revision. Secondary rotator cuff tear was the most common complication after ATSA, occurring in 3.7% of patients, but only 1.1% of patients required revision surgery. Both ATSA and RTSA implant survivorship was reported to range from 93.1% to 98.9% at 5- and 8-year follow-up, respectively. Patient mortality was estimated to be 19.3% with a mean time to death of 6.1 years.

**Conclusions:**

Elderly patients with primary osteoarthritis and an intact rotator cuff can have predictable pain relief, restoration of functional range of motion, and significant improvement in PROs after ATSA with low complication rates. Secondary rotator cuff failure and revision arthroplasty occur infrequently at early to mid-term follow-up. Although elderly patients who underwent ATSA generally had better functional outcomes compared to those who underwent RTSA for differing indications, patient satisfaction after both procedures were similar.

Total shoulder arthroplasty (TSA) utilization continues to increase worldwide due to an aging population, with the demand for primary arthroplasty projected to increase by 333% from 2011 to 2030.^31^ This trend is most evident in the elderly, for whom the incidence has increased at the greatest rate.^40^ Both anatomic total shoulder arthroplasty (ATSA) and reverse total shoulder arthroplasty (RTSA) are acceptable treatment options for multiple distinct shoulder pathologies with reliable improvements in pain and function.^28^^,^^32^^,^^36^^,^^39^ Despite this, there remain concerns about these procedures among elderly patients due to their general health, the potential for lesser functional gain, worse bone quality due to osteopenia, the potential for postoperative instability due to compromised soft tissues, and the possible need for revision at an even older age.^11^^,^^15^ Therefore, shoulder arthroplasty, particularly in patients over 70 years, is a topic of growing interest.

ATSA has historically been indicated for patients with primary glenohumeral osteoarthritis (OA) and a functioning rotator cuff, whereas the more highly-constrained RTSA has been reserved for patients with glenohumeral arthritis without an intact or functioning rotator cuff. However, reliable clinical results of RTSA have expanded the indications to include other conditions such as OA with biconcave glenoids, massive irreparable rotator cuff tears, proximal humerus fractures (PHFs), fracture sequelae, inflammatory arthropathy, tumor, and revision arthroplasty.^46^ More recently, several studies have even recommended considering RTSA over the traditional ATSA for elderly patients with primary OA and an intact rotator cuff.^12^^,^^16^ This suggestion is largely due to concerns about rotator cuff integrity and dysfunction related to disuse in elderly patients undergoing TSA.^44^^,^^46^ In fact, recent utilization rates of RTSA for OA have demonstrated a significant upward trend from 15% to 29% over the past decade.^9^

To date, there have been multiple studies that have evaluated the outcomes of ATSA or RTSA in patients over the age of 70. These studies, however, consisted of small patient numbers with variable follow-up, resulting in limited evidence to guide treatment in this population. Additionally, there have been no systematic reviews that have reported on this important topic. Therefore, the purpose of this systematic review is to compare the clinical outcomes, radiographic outcomes, and complications of ATSA and RTSA among patients older than 70 years.

## Methods

### Search strategy

**Figure 1:**
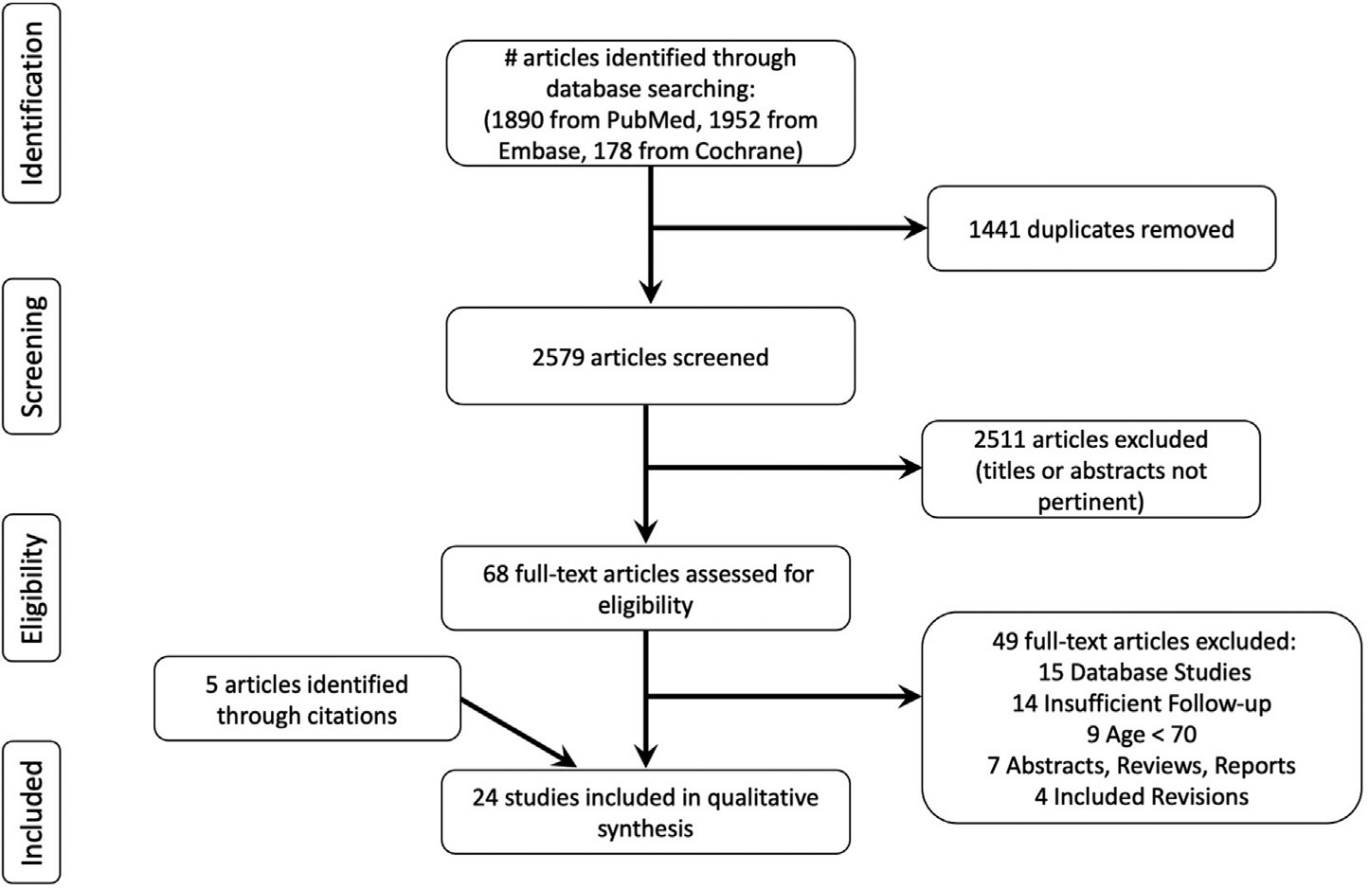
The Preferred Reporting Items for Systematic Reviews and Meta-Analyses diagram of the literature search and study selection.

A systematic review was performed using guidance from the checklist of the Preferred Reporting Items for Systematic Reviews and Meta-Analyses (PRISMA) (Fig. 1). A search was conducted using PubMed, Embase, and Cochrane Central Register of Controlled Trial databases on December 31, 2022, with the terms: (“total shoulder arthroplasty” or “total shoulder replacement” or “reverse shoulder arthroplasty” or “reverse shoulder replacement”) and (“older” or “elderly” or “elder” or “70” or “75” or “80”). A total of 2579 articles were identified after removal of duplicates. The inclusion criteria were studies with patients older than 70 years who were treated with a primary ATSA or RTSA and clinical results reported at a minimum of 2 years. All indications for primary shoulder arthroplasty except for tumor were included. The exclusion criteria were as follows: abstracts, case reports, nationwide database studies, literature reviews, and biomechanical studies; studies involving heterogeneous treatments (eg, hemiarthroplasty and TSA) without separately reporting outcomes; studies with results mixed with patients younger than 70 years; and studies with results mixed with revision arthroplasty.

Two authors (F.S. and P.N.) independently screened the titles, abstracts, and full texts. Any discrepancies in inclusion or exclusion were carried to the next round of screening to ensure thoroughness. References to each included study were further screened to capture any publications that may have eluded the original search queries. A total of 24 articles were included for review.

### Assessment of study quality

Two authors (F.S. and P.N.) independently assessed the methodological quality of all included studies with the methodological index for non-randomized studies (MINORS), which is a validated instrument designed to assess the methodological quality of nonrandomized surgical studies.^41^ For noncomparative studies, 8 items were evaluated from 0 to 2, leading to a maximum score of 16. For comparative studies, 4 additional items were included, with a maximum total score of 24. All discrepancies between reviewers were resolved by consensus.

### Outcomes

Relevant data were extracted, including patient demographics, diagnosis, type of replacement, complications, revisions, clinical outcomes (mortality, satisfaction, and range of motion), patient-reported outcomes (PROs), and radiologic outcomes at last follow-up. Only studies that clearly reported postoperative complication, reoperation, or revision data were included in the pooled analysis of complications and revisions. For major complications, such as infection, instability, rotator cuff tear, and periprosthetic fracture, it was assumed that these complications did not occur unless they were explicitly reported to have happened. However, for component loosening and minor complications, such as acromial stress fracture, nerve injury, and hematoma, studies that did not specifically mention these complications were excluded from the pooled analysis. Data were aggregated by type of arthroplasty. Weighted mean estimates and ranges were provided for clinical outcomes, radiological outcomes, complications, and revisions.

## Results

Twenty-four studies were included in this review (Table I). Eleven studies reported the outcomes of ATSA with the primary diagnosis of glenohumeral OA in all patients. Twenty studies reported the outcomes of RTSA with 8 studies focused solely on acute PHF.^6^^,^^10^^,^^13^^,^^17^^,^^20^^,^^25^^,^^34^^,^^35^ Of the other 12 RTSA studies, 7 had varied diagnoses,^2^^,^^4^^,^^11^^,^^14^^,^^26^^,^^27^^,^^39^ whereas 2 studies included only glenohumeral OA^28^^,^^46^ and 3 studies included only rotator cuff arthropathy.^32^^,^^36^^,^^44^ Seven studies compared the outcomes of ATSA to RTSA.^2^^,^^28^^,^^32^^,^^36^^,^^39^^,^^44^^,^^46^ Six studies compared the outcomes of an older cohort to a younger cohort.^4^^,^^18^^,^^27^^,^^32^^,^^36^^,^^38^ Seven studies analyzed the outcomes of patients over the age of 80 years.^4^^,^^11^^,^^14^^,^^15^^,^^18^^,^^38^^,^^44^ The MINORS score for noncomparative studies was 9.2, which indicates a low quality of evidence. The MINORS score for comparative studies was 16.7, which indicates a moderate level of evidence.

**Table I:** Study characteristics according to arthroplasty type.

	No. of studies	Level of evidence	No. of patients	Follow-up (yr)	Age (yr)	Female	Diagnosis	Mortality
ATSA	11	III: 8, IV: 3	926	3.4 (2.0-6.7)	76.9 (74.0-84.0)	60.1%	OA: 100%	116/507 (22.9%)
RTSA								
No fracture	12	III: 10, IV: 2	1067	3.1 (2.0-4.9)	82.0 (75.0-87.2)	75.4%	RCA: 70.3%, OA: 17.3%, MIRCT: 4.1%	67/439 (15.3%)
Fracture	8	I: 2, II: 1, III: 3, IV: 2	310	2.7 (2.4-3.1)	78.8 (74.7-81.8)	87.0%	PHF: 100%	18/179 (10.1%)

*ATSA*, anatomic total shoulder arthroplasty; *RTSA*, reverse total shoulder arthroplasty; *OA*, osteoarthritis; *RCA*, rotator cuff arthropathy; *MIRCT*, massive irreparable rotator cuff tear; *PHF*, proximal humerus fracture.

Follow-up, age, sex, and diagnosis represent weighted mean values by sample size; ranges reported in parentheses. Only the three most common diagnoses are listed.

### Patient-reported outcomes

There were 12 different outcome scores reported after ATSA with American Shoulder and Elbow Surgeons (ASES), Constant, and visual analog scale (VAS) pain scores being the most common (Table II). The ASES score improved from 38 to 84 at a mean follow-up of 3.2 years. The Constant Score improved from 28 to 76 at a mean follow-up of 3.1 years, and VAS pain scores decreased from 6.5 to 1.1 at a mean follow-up of 3.4 years. Overall, 90.9% of patients in 6 studies reported to be satisfied with the ATSA procedure.^15^^,^^18^^,^^32^^,^^39^^,^^44^^,^^46^

**Table II:** Weighted means for patient-reported outcomes according to arthroplasty type.

	VAS pain	ASES	Constant	Satisfaction
ATSA				
No. of studies	8	6	6	6
No. of patients	677	685	363	398
Preoperative	6.5 (5.6-9.8)	38 (37-40)	28 (25-54)	NA
Follow-up	1.1 (0.0-2.0)	84 (78-91)	76 (65-89)	90.9% (80.0%-100%)
Change	5.4 (4.9-7.8)	46 (41-49)	48 (35-51)	NA
RTSA (no fracture)				
No. of studies	10	8	7	6
No. of patients	898	690	696	551
Preoperative	6.6 (5.7-9.0)	35 (32-41)	29 (16-39)	NA
Follow-up	1.0 (0.0-1.6)	79 (73-83)	64 (56-80)	90.8% (70.0%-93.0%)
Change	5.6 (4.5-8.0)	44 (38-46)	35 (32-46)	NA
RTSA (fracture)				
No. of studies	4	4	7	2
No. of patients	178	161	288	61
Follow-up	1.2 (0.9-1.5)	74 (72-77)	61 (47-76)	88.6% (87.0%-91.0%)

*VAS*, Visual Analog Scale; *ASES*, American and Shoulder Elbow Surgeons; *ATSA*, anatomic total shoulder arthroplasty; *RTSA*, reverse total shoulder arthroplasty; *NA*, not applicable.

Ranges reported in parentheses.

Fourteen different outcome scores were reported after RTSA for nonfracture indications with ASES, Constant, VAS pain, and Shoulder Pain and Disability Index (SPADI) scores being the most common (Table II). The ASES score improved from 35 to 79 at a mean follow-up of 3.4 years. Similarly, the Constant Score improved from 29 to 64 at a mean of 3.3 years. VAS pain scores decreased from 6.6 to 1.0 at a mean follow-up of 3.5 years, and Shoulder Pain and Disability Index decreased from 84 to 26 at a mean follow-up of 3.4 years. The satisfaction rate in 6 studies was 90.8%.^4^^,^^26^^,^^39^^,^^44^^,^^46^

There were 11 different outcome measures used to evaluate PROs of RTSA performed for PHF, with ASES, Constant, and VAS pain scores being the most common. The ASES, Constant, and VAS pain scores were 74, 61, and 1.2, respectively, at a mean follow-up of 2.7 years. Overall, 88.6% of patients in 2 studies were satisfied with the outcome of the procedure.^6^^,^^13^

Among 4 ATSA studies including only patients over 80 years,^15^^,^^18^^,^^38^^,^^44^ the mean ASES score improved from 38 to 91 at 3.3 years in 1 study,^44^ and the mean Constant Score improved from 38 to 75 at 3.0 years in 2 studies.^18^^,^^38^ In 4 RTSA studies among patients over 80 years, ASES score increased from 35 to 81, Constant Score increased from 30 to 63, and VAS pain decreased from 6.7 to 1.1 at 3.2 years.^4^^,^^44^

There were 2 studies that evaluated the clinical outcomes of RTSA in patients with glenohumeral OA and an intact rotator cuff.^28^^,^^46^ The ASES score was 83 at 4.8 years,^46^ and the Constant Score was 68 at 2.4 years.^28^ One study reported that the Constant Score was significantly higher in the ATSA group compared to that of the RTSA group.^28^ However, no difference in ASES score was observed between ATSA and RTSA groups in another study.^46^

### Range of motion

Eight studies reported both pre-and postoperative motion after TSA.^2^^,^^15^^,^^18^^,^^19^^,^^28^^,^^38^^,^^39^^,^^44^ There was a mean improvement from 93° to 153° in forward elevation, 96° to 129° in abduction, and 20° to 55° in external rotation (Table III). In studies reporting internal rotation relative to vertebral level, internal rotation improved a mean of 5 vertebral levels.^2^^,^^15^^,^^28^^,^^38^

**Table III:** Weighted means of range of motion according to arthroplasty type.

	Flexion (°)	Abduction (°)	External rotation (°)
ATSA			
No. of studies	8	3	8
No. of patients	476	88	476
Preoperative	93 (80-105)	96 (70-103)	20 (7-26)
Follow-up	153 (137-170)	129 (90-160)	55 (23-64)
Change	60 (38-65)	33 (20-58)	35 (13-43)
RTSA (No Fracture)			
No. of studies	8	6	8
No. of patients	804	593	804
Preoperative	71 (60-93)	74 (60-106)	18 (8-26)
Follow-up	130 (90-160)	129 (90-160)	36 (20-47)
Change	59 (30-77)	57 (19-77)	18 (5-30)
RTSA (fracture)			
No. of studies	8	5	7
No. of patients	312	213	281
Follow-up	125 (118-139)	112 (109-114)	22 (17-24)

*ATSA*, anatomic total shoulder arthroplasty; *RTSA*, reverse total shoulder arthroplasty.

External rotation measured with arm at side. Ranges reported in parentheses.

Among RTSA studies performed for nonfracture indications, 8 studies reported both pre- and postoperative motion.^2^^,^^4^^,^^11^^,^^14^^,^^27^^,^^28^^,^^39^^,^^44^ Forward elevation improved from 71° to 130°, whereas abduction improved from 74° to 129° and external rotation improved from 18° to 36° (Table III). Internal rotation was measured in 7 studies with mean improvement of 2 vertebral levels.^2^^,^^4^^,^^11^^,^^14^^,^^27^^,^^28^^,^^46^

For RTSA performed for acute PHF, postoperative forward elevation, abduction, and external rotation were 125°, 112°, and 22°, respectively (Table III). Two studies achieved a mean internal rotation level of L4 level,^10^^,^^34^ whereas 2 others reported attaining between L4 and L5 or buttocks and sacrum.^6^^,^^35^

Subgroup analysis of ATSA studies including only patients over 80 years showed that forward elevation improved from 87° to 141° and external rotation improved from 15° to 43°.^15^^,^^18^^,^^38^^,^^44^ Similarly, forward elevation improved from 68° to 129° and external rotation improved from 18° to 37° in patients over 80 years who underwent RTSA for nonfracture indications.^4^^,^^11^^,^^14^^,^^44^

Among RTSA patients with a diagnosis of glenohumeral OA and an intact rotator cuff, flexion, abduction, and external rotation at final follow-up was 160°, 150°, and 20°, respectively.^28^ One study reported that abduction was significantly higher in the ATSA group compared to that of the RTSA group,^28^ though no significant difference in motion was observed between groups in another study.^46^

### Radiological outcomes

Seven TSA studies evaluated radiological outcomes at a mean follow-up of 3.2 years (Table IV).^2^^,^^15^^,^^18^^,^^19^^,^^28^^,^^38^^,^^39^ On the humeral side, long-stem implants were used in 3 studies,^15^^,^^18^^,^^19^ short-stem implants in 2 studies,^2^^,^^28^ stemless implants in 1 study,^39^ and mixed implants in 1 study.^38^ All but 1 study predominantly utilized press-fit implants, except when bone quality was poor and cement was utilized.^15^^,^^38^ Humeral radiolucencies were assessed with 3 different methods and were present in 1.4% of patients. There was only 1 (0.2%) case of humeral loosening that had no reported clinical consequence.^15^ Humeral subluxation in TSA was reported in 3 studies with a 7.8%, 8.3%, and 2.3% rate of superior, anterior, and posterior subluxation, respectively.^15^^,^^18^^,^^19^ One study found that patients with moderate to severe superior humeral migration had worse Constant and pain scores compared to patients with no or mild migration;^18^ however, the correlation between humeral subluxation and PROs was not corroborated by other studies.^15^^,^^19^

**Table IV:** Radiological evaluation of anatomic and reverse total shoulder arthroplasty.

	ATSA			RTSA (no fracture)			RTSA (fracture)		
	No. of Studies	No. of pooled patients	Rate	No. of studies	No. of pooled patients	Rate	No. of studies	No. of Pooled Patients	Rate
Humeral radiolucency	6	423	1.4% (0%-9.1%)	5	559	7.2% (2.3%-16.3%)	5	205	4.9% (0%-12.9%)
Humeral loosening	7	471	0.2% (0%-2.1%)	6	383	0.3% (0%-0.6%)	6	229	0% (no range)
Humeral subluxation									
Superior	3	348	7.8% (3.1%-10.4%)	NA	NA	NA	NA	NA	NA
Anterior	3	348	8.3% (4.2%-9.4%)	NA	NA	NA	NA	NA	NA
Posterior	3	348	2.3% (0%-3.1%)	NA	NA	NA	NA	NA	NA
Glenoid radiolucency	5	182	42.9% (13.6%-100%)	3	254	2.4% (0%-9.4%)	3	109	0% (no range)
Glenoid loosening	7	471	3.6% (0%-18.8%)	6	383	0.5% (0%-1.1%)	6	229	0% (no range)
Scapular notching	NA	NA	NA	7	645	18.6% (6.3%-41.1%)	8	308	20.5% (0%-47.4%)
Anatomic tuberosity union	NA	NA	NA	NA	NA	NA	8	308	65.6% (36.8%-84.2%)

*ATSA*, anatomic total shoulder arthroplasty; *RTSA*, reverse total shoulder arthroplasty; *NA*: not applicable.

For each radiological outcome, the rate is calculated relative to the total number of patients within the pooled data. The range of rates for the included studies is reported in parentheses.

All ATSA studies used a cemented pegged or keeled glenoid all-polyethylene glenoid component. However, in 1 study, 40% of their patients had received a metal-backed glenoid component.^15^ Glenoid radiolucencies were reported in 5 studies with a prevalence of 42.9% using variable classification systems. Of which, 3.6% of patients were found to have radiologically loose glenoid components, but none underwent revision surgery as 24% of these patients declined further surgery and the remaining patients did not have clinically relevant symptoms.^15^^,^^18^ The presence of a glenoid radiolucency was not associated with pain or range of motion in 1 study.^15^

Seven RTSA studies for nonfracture indications assessed the radiological outcome at a mean of 3.0 years (Table IV).^4^^,^^11^^,^^14^^,^^26^^,^^27^^,^^28^^,^^39^ Long-stem implants were used in 4 studies,^11^^,^^26^^,^^27^^,^^39^ short-stem implants in 1 study,^28^ mixed implants in 1 study,^14^ and the remaining study did not specify implant used.^4^ Humeral radiolucencies were identified in 7.2% of patients, but radiologically loose humeral components were found in only 1 (0.3%) patient^11^ Glenoid radiolucencies were reported only in 3 studies and found in 2.4% of patients.^11^^,^^27^^,^^28^ Radiological baseplate loosening was identified in 2 (0.5%) patients, of which only 1 patient required revision surgery.^11^ Scapular notching was reported in all studies using the Sirveaux-Nerot classification and was present in 18.6% of shoulders. Of the 4 studies that graded the severity of scapular notching, 16.3% was grade 1, 6.2% grade 2, 2.1% grade 3, and 0.6% grade 4.^11^^,^^27^^,^^28^

Among 8 RTSA studies for PHF, radiological assessment was performed at a mean follow-up of 2.7 years.^6^^,^^10^^,^^13^^,^^17^^,^^20^^,^^25^^,^^34^^,^^35^ Humeral radiolucencies were present in 4.9% of patients. No glenoid radiolucencies were identified and there was no radiological loosening of either component. Scapular notching was present in 20.5% of shoulders, but of the 6 studies that graded the severity of notching, 9.6% was grade 1, 7.0% grade 2, and 1.3% grade 3.^10^^,^^13^^,^^17^^,^^20^^,^^25^^,^^35^ Anatomic healing of the tuberosity occurred in 65.6% of shoulders.

### Complications

The overall complication rate of 9 ATSA studies was 10.2% (range, 3.8%-28.9%) (Table V).^2^^,^^15^^,^^18^^,^^19^^,^^28^^,^^38^^,^^39^^,^^44^^,^^46^ The most common complication was secondary rotator cuff tear, which occurred in 3.7% of patients. Other less common complications included glenoid loosening (2.9%), hematoma (1.1%), nerve injury (0.9%), intraoperative fracture (0.7%), instability (0.6%), periprosthetic fracture (0.4%), and prosthetic joint infection (0.1%). The only prosthetic joint infection was treated with long-term suppressive antibiotics without surgery.^19^

**Table V:** Complications and reoperations after anatomic and reverse total shoulder arthroplasties.

	ATSA			RTSA (no fracture)			RTSA (fracture)		
	No. of Studies	No. of pooled patients	Rate	No. of studies	No. of pooled patients	Rate	No. of studies	No. of pooled patients	Rate
Complications									
Overall	9	697	10.2% (3.8%-28.9%)	10	907	9.9% (3.5%-41.9%)	8	313	5.4% (0%-10.3%)
Rotator cuff tear	9	697	3.7% (0%-11.1%)	NA	NA	NA	NA	NA	NA
Acromial stress fracture	NA	NA	NA	6	441	6.1% (2.3%-25.6%)	2	79	0% (no range)
Glenoid loosening	7	577	2.9% (0%-18.8%)	7	729	0.3% (0%-0.6%)	6	234	0% (no range)
Intraoperative fracture	9	697	0.7% (0%-2.2%)	10	907	0.6% (0%-6.3%)	8	313	0% (no range)
Periprosthetic fracture	9	697	0.4% (0%-2.0%)	10	907	1.0% (0%-3.2%)	8	313	1.0% (0%-4.2%)
Instability	9	697	0.6% (0%-1.0%)	10	907	0.9% (0%-3.0%)	8	313	0.6% (0%-2.4%)
Nerve injury	5	577	0.9% (0%-14.3%)	5	389	3.1% (1.1%-3.1%)	4	154	1.9% (0%-4.2%)
Prosthetic joint infection	9	697	0.1% (0%-0.3%)	10	907	0.4% (0%-3.1%)	8	313	1.0% (0%-3.4%)
Hematoma	4	556	1.1% (0.8%-3.1%)	3	244	0.4% (0%-3.1%)	5	193	2.6% (0%-4.8%)
Reoperation									
Overall	10	905	2.3% (0%-6.9%)	11	1011	2.2% (0%-7.7%)	8	313	3.2% (0%-9.5%)
Revision arthroplasty	10	905	2.0% (0%-6.7%)	11	1011	1.4% (0%-6.7%)	8	313	1.6% (0%-5.2%)

*ATSA*, anatomic total shoulder arthroplasty; *RTSA*, reverse total shoulder arthroplasty; *NA*, not applicable.

For each complication or revision, the rate is calculated relative to the total number of patients within the pooled data. The range of rates for the included studies is reported in parentheses.

Among 10 RTSA studies for nonfracture indications, the total complication rate was 9.9% (range, 3.5%-41.9%) (Table V).^2^^,^^4^^,^^11^^,^^14^^,^^26^^,^^27^^,^^28^^,^^39^^,^^44^^,^^46^ The most common complication was an acromion stress fracture, occurring in 6.1% of patients. Other less common complications included nerve injury (3.1%), periprosthetic fracture (1.0%), instability (0.9%), intraoperative fracture (0.4%), and prosthetic joint infection (0.4%). All nerve injuries except for 3 patients resolved spontaneously at the time of final follow-up.^2^^,^^11^^,^^14^^,^^44^^,^^45^

For 8 RTSA studies performed acute PHF, the total complication rate was 5.4% (range, 0%-10.3%).^6^^,^^10^^,^^13^^,^^17^^,^^20^^,^^25^^,^^34^^,^^35^ The most common complication was postoperative hematoma, which occurred in 2.6% of patients. Other less common complications included nerve injury (1.9%), prosthetic joint infection (1.0%), periprosthetic humerus fracture (1.0%), and instability (0.6%).

### Reoperations and implant survivorship

Ten TSA studies reported reoperation rate of 2.3% (range, 0%-6.9%) at 3.7 years (Table V).^2^^,^^15^^,^^18^^,^^19^^,^^28^^,^^32^^,^^38^^,^^39^^,^^44^^,^^46^ The most common indication for reoperation after TSA was secondary rotator cuff tear. Although 3.7% of patients had secondary rotator cuff tears, only 1.1% of patients underwent revision arthroplasty for this indication. Three studies reported that the reason that over half of their patients chose to forgo revision surgery was due to minimal pain and maintenance of sufficient mobility to perform activities of daily living.^15^^,^^19^^,^^39^ Less common causes for revision arthroplasty included instability (0.4%), periprosthetic fracture (0.3%), and glenoid loosening (0.1%). In the 2 studies that reported complications of glenoid loosening, all patients declined further surgery due to mild clinical symptoms.^15^^,^^18^ Two studies reported the reoperation-free implant survival with 98.9% of implants surviving at 5-year and 93.1% of implants surviving at 8-year follow-ups.^19^^,^^46^

The total reoperation rate among RTSA performed for nonfracture indications was 2.2% (range, 0%-7.7%) at 3.2 years (Table V).^2^^,^^4^^,^^11^^,^^14^^,^^26^^,^^27^^,^^28^^,^^32^^,^^39^^,^^44^^,^^46^ Prosthetic joint infection was the most common indication for revision arthroplasty, occurring in 0.6% of patients. All infections were treated in two-stage fashion. Less common procedures included revision arthroplasty for instability (0.5%), open reduction internal fixation of a periprosthetic humerus fracture (0.4%), hematoma evacuation (0.1%), and open reduction (0.1%) for instability. Two studies reported the reoperation-free implant survival with 98.3% of implants surviving at 5-year and 97% of implants surviving at 8-year follow-ups.^11^^,^^46^

At 2.7-year follow-up, the reoperation rate of RTSA performed for acute PHF was 3.2% (range, 0%-9.5%).^6^^,^^10^^,^^13^^,^^17^^,^^20^^,^^25^^,^^34^^,^^35^ Hematoma evacuation and prosthesis explant performed for a deep infection were the most common procedures, both occurring in 1.0% of patients. Additionally, there were 2 revision arthroplasties performed for instability. Implant survivorship was reported in only 1 study with a 96.8% survival at 3.3 years.^35^

### Mortality

Among ATSA and RTSA studies for nonfracture indications, 7 studies reported mortality after surgery.^11^^,^^14^^,^^15^^,^^19^^,^^26^^,^^38^^,^^46^ Three studies utilized the Mayo Clinic Total Joint registry, though there was no overlap in the study period or the type of arthroplasty.^11^^,^^15^^,^^19^ The mortality rate after surgery was 19.3% with 3 studies reporting a mean time to death of 6.1 years.^11^^,^^15^^,^^46^ In 1 study that analyzed patients over 80 years using the Mayo Clinic Total Joint Registry, the mortality rate was 87% after ATSA at a mean time of 7.5 years.

Among PHF studies, there were 4 studies that reported mortality after surgery.^6^^,^^20^^,^^25^^,^^35^ The mortality rate was 10.1% with all patient deaths occurred within 3 years of surgery. The mean time to death was reported in one study and was 1.6 years.^25^ One patient who underwent RTSA for a PHF died 8 days after surgery secondary to pneumonia.^20^ No other deaths were related to the procedure.

## Discussion

In this systematic review of 24 studies including patients older than 70 years, TSA is associated with excellent clinical outcomes and low complication rates at early to mid-term follow-up. Secondary rotator cuff tears and revisions were rare despite the mean age of 77 years. Moreover, patients who underwent ATSA generally had better motion and functional outcomes compared to those who underwent RTSA; however, these comparisons were made across different indications for arthroplasty. Similar trends in clinical outcomes were also observed among studies including only patients older than 80 years.

Although both prostheses had significant improvements in all planes of motion, the final postoperative forward elevation, external rotation, and internal rotation were 23°, 19°, and 3 vertebral levels greater in patients who underwent ATSA compared to those who underwent RTSA for nonfracture indications, respectively. These findings may reflect the absence of a functional rotator cuff in the majority of patients who underwent RTSA for rotator cuff arthropathy and the decreased moment arms of the rotator cuff muscles associated with the medialized center of rotation in a reverse prostheses.^3^ Additionally, technical variation in the glenosphere position and humeral offset may also contribute to the decreased active motion seen in RTSA studies, as maximal impingement-free range of motion has been shown to occur with increased inferior translation, inferior tilt, and lateralization of the glenosphere.^24^ In a subgroup analysis of ATSA and RTSA performed for OA with an intact cuff, the results were mixed with one study showing superior motion among ATSA patients and another showing no difference.^28^^,^^46^ A recent meta-analysis of 6 studies including patients of all ages with OA and an intact cuff found that external rotation was significantly better for ATSA than for RTSA.^22^ Elderly patients who underwent RTSA for acute PHF also had less external rotation than patients who underwent RTSA for nonfracture indications. Nonunion or malunion of the greater tuberosity occurred in 34.4% of patients and has been shown to be associated with decreased flexion and external rotation.^6^^,^^10^

Despite starting with similar preoperative ASES and Constant Scores, patients over the 70 years who underwent ATSA had higher absolute and change in scores compared to those who underwent RTSA for nonfracture indications. Shah et al^36^ reported that the percentage of patients over 75 years achieving minimal clinically important difference was similar between ATSA and RTSA, but 90.5% of ATSA patients achieved substantial clinical benefit compared to 76.9% of RTSA patients. Additionally, only 1 of 6 studies that directly compared ATSA and RTSA in the elderly reported no significant differences.^46^ The improved outcomes among TSA patients may be attributed to higher gains in motion and function rather than decreased pain, as VAS pain scores were similar in both groups before and after surgery. It should be noted, however, that many of the patients undergoing RTSA had very distinctly different pathologic diagnoses and would not be appropriate candidates for ATSA given the lack of a functional rotator cuff. Of the 2 RTSA studies in this review that only included patients with OA and an intact cuff, the Constant Score was lower than that of the pooled ATSA patients, but the ASES score was similar.^28^^,^^46^ Kim et al^22^ also found no significant difference in Constant Score, ASES, subjective shoulder value, and Simple Shoulder Test in a meta-analysis of ATSA and RTSA studies performed for OA with an intact cuff. While there appears to be an advantage of ATSA over RTSA in terms of functional outcomes, the percentage of patients who were satisfied after the surgery was similar at 90.9% and 91.3%, respectively. These satisfaction rates are consistent with what has been reported for all ages in the literature, which ranges from 75% to 100% for ATSA and 79% to 90% for RTSA.^33^

Concerns about late rotator cuff failure after ATSA coupled with reliable satisfactory outcomes after RTSA for multiple indications has prompted some surgeons to utilize RTSA for elderly patients with glenohumeral OA with an intact rotator cuff.^16^ Although secondary symptomatic rotator cuff tears after ATSA were the most common complication and indication for revision, the overall incidence was low. In this review, 3.7% of patients were found to have secondary rotator cuff tear with only 1.1% of patients requiring revision surgery. Patients who declined revision had minimal pain and maintenance of acceptable function to perform activities of daily living.^15^^,^^19^^,^^39^ It is also important to consider how one defines secondary rotator cuff failure as this can influence the reported complication rate. Prior studies have used radiographic evidence of postoperative superior humeral head migration as the definition of secondary rotator cuff tear, regardless of patient symptoms.^45^^,^^47^ For example, in a series of 518 patients at 8.6 year follow-up, Young et al^47^ identified radiographic evidence of rotator cuff dysfunction in 16.8% of patients, yet only 1 patient went onto revision due to cuff failure. Utilizing this definition could potentially overestimate the cuff failure rate and would increase its incidence to 7.8% in this review.

The surgical complication rates of ATSA and RTSA in patients over 70 years were similar and low at 10.2% and 9.9%, respectively. These findings contrast with older systematic reviews that reported a much higher complication rate ranging from 16.1% to 24.0% after RTSA at a mean follow-up of 3.5 years, suggesting that complications have decreased due to contemporary surgical techniques and implants.^5^^,^^48^ Not surprisingly, the most common complication after RTSA in our review was an acromion stress fracture, which occurred in 6.1% of patients. This incidence is higher than that of prior studies, which ranged from 1.5% to 2.6%.^37^^,^^48^ The increased frequency of these insufficiency fractures may have been compounded by the decreased bone quality among our older population.^1^ The consequences of acromial stress fractures have been demonstrated in several prior studies which showed a consistent trend of decreased PROs compared to those who did not sustain a fracture.^23^^,^^43^ Furthermore, the overall reoperation rate among our elderly population was low at 2.3% at 3.7 years for ATSA and 2.2% at 3.2 years for RTSA. These rates are comparable to data from the New Zealand Joint Registry which reported rates of 3.2% for ATSA and 1.7% for RTSA in patients over 70 years.^29^ Additionally, the reoperation-free implant survivorship in patients over 70 years was 95% and 98% for TSA and RTSA at 14 years, respectively.^29^ Collectively, these findings suggest that both ATSA and RTSA are predictable and reliable procedures among elderly patients.

Shoulder arthroplasty in this elderly population was relatively safe with only 2 deaths (0.1%) within 90 days of surgery despite high prevalence of comorbidities. The 30-day and 90-day mortality of octogenarians in national database studies have been reported to be between 0.22% and 0.5% and 2.7%, respectively.^7^^,^^8^^,^^42^ The overall mortality of all included studies was 19.3%, which is not surprising considering that the mean age of included studies was 79.5 years and the current life expectancy in the United States is 74.2 years for men and 79.9 years for women.^30^ Given excellent implant survivorship of both ATSA and RTSA over 98% at 5 years, it is probable that most patients over 70 years will not need a revision procedure.^11^^,^^19^

There were several limitations to this review. First, RTSA studies for differing indications were aggregated together. Although an attempt was made to separate fractures and primary OA with an intact cuff from other degenerative indications, a recent systematic review demonstrated that the clinical outcomes and complications of RTSA vary by preoperative diagnoses.^21^ High quality studies comparing the outcomes of ATSA and RTSA for glenohumeral OA with an intact cuff are certainly warranted. Additionally, the implants and technical aspects of the procedure, such as preparation of the glenoid and humerus, management of bone loss, and cementation of components, were inconsistently reported and of varying quality. Factors that may modify the amount of impingement-free range of motion, including the type RTSA prosthesis design (medial glenoid/medial humerus, medial glenoid/lateral humerus, and lateral glenoid/medial humerus), degree of inferior tilt, and amount of inferior translation, could not be consistently determined. As such, no definitive conclusions could be reached about the impact of these practices on motion, outcomes, component loosening, or revision rates. Furthermore, important preoperative imaging parameters, such as glenoid classification, degree of retroversion, and rotator cuff muscle quality, were not included in data extraction due to sporadic reporting. Lastly, the utilization of over 10 different PRO instruments among studies limited the ability to represent some studies when aggregating data.

## Conclusions

Elderly patients with primary OA and an intact rotator cuff can have predictable pain relief, restoration of functional range of motion, and significant improvement in PROs after ATSA with low complication rates. Secondary rotator cuff failure and revision arthroplasty occur infrequently at early to mid-term follow-up. Elderly patients who underwent ATSA generally had better functional outcomes compared to those who underwent RTSA for differing indications, but patient satisfaction after both procedures were similar. Despite recent trends, age greater than 70 years should not be the sole reason for selecting RTSA over ATSA in patients with primary OA.

## Disclaimers:

Funding: Research reported in this publication was supported by 10.13039/100000049National Institute on Aging grant number R38AG070171. Its contents are solely the responsibility of the authors and do not necessarily represent the official views of the National Institute of Health.

Conflicts of interest: The authors, their immediate families, and any research foundation with which they are affiliated have not received any financial payments or other benefits from any commercial entity related to the subject of this article.

## References

[1] Mahendraraj K.A., Abboud J., Armstrong A., Austin L., Brolin T., ASES Complications of RSA Research Group (2021). Predictors of acromial and scapular stress fracture after reverse shoulder arthroplasty: a study by the ASES Complications of RSA Multicenter Research Group. J Shoulder Elbow Surg.

[2] Barret H., Bonnevialle N., Azoulay V., Baron-Trocellier T., Mansat P. (2021). Short-stem uncemented anatomical shoulder replacement for osteoarthritis in patients older than 70 years: is it appropriate?. JSES Int.

[3] Berliner J.L., Regalado-Magdos A., Ma C.B., Feeley B.T. (2015). Biomechanics of reverse total shoulder arthroplasty. J Shoulder Elbow Surg.

[4] Boettcher M.L., Neel G.B., Reid J.J., Eichinger J.K., Friedman R.J. (2022). Clinical and radiographic outcomes after reverse total shoulder arthroplasty in patients 80 years of age and older. J Shoulder Elbow Surg.

[5] Bohsali K.I., Bois A.J., Wirth M.A. (2017). Complications of shoulder arthroplasty. J Bone Joint Surg Am.

[6] Boileau P., Alta T.D., Decroocq L., Sirveaux F., Clavert P., Favard L. (2019). Reverse shoulder arthroplasty for acute fractures in the elderly: is it worth reattaching the tuberosities?. J Shoulder Elbow Surg.

[7] Bovonratwet P., Malpani R., Ondeck N.T., Tyagi V., Grauer J.N. (2019). Elective total shoulder arthroplasty in octogenarians: a safe procedure. J Am Acad Orthop Surg.

[8] Carney J., Gerlach E., Plantz M.A., Cantrell C., Swiatek P.R., Marx J.S. (2021). Short-term outcomes after total shoulder arthroplasty in octogenarians: a matched analysis. Cureus.

[9] Chalmers P.N., Salazar D.H., Romeo A.A., Keener J.D., Yamaguchi K., Chamberlain A.M. (2018). Comparative utilization of reverse and anatomic total shoulder arthroplasty: a comprehensive analysis of a high-volume center. J Am Acad Orthop Surg.

[10] Chun Y.M., Kim D.S., Lee D.H., Shin S.J. (2017). Reverse shoulder arthroplasty for four-part proximal humerus fracture in elderly patients: can a healed tuberosity improve the functional outcomes?. J Shoulder Elbow Surg.

[11] Clark N.J., Samuelsen B.T., Alentorn-Geli E., Assenmacher A.T., Cofield R.H., Sperling J.W. (2019). Primary reverse shoulder arthroplasty in patients older than 80 years of age: survival and outcomes. Bone Joint J.

[12] Collin P., Herve A., Walch G., Boileau P., Muniandy M., Chelli M. (2019). Mid-term results of reverse shoulder arthroplasty for glenohumeral osteoarthritis with posterior glenoid deficiency and humeral subluxation. J Shoulder Elbow Surg.

[13] Cuff D.J., Pupello D.R. (2013). Comparison of hemiarthroplasty and reverse shoulder arthroplasty for the treatment of proximal humeral fractures in elderly patients. J Bone Joint Surg Am.

[14] Endell D., Audige L., Imiolczyk J.P., Scheibel M., Freislederer F. (2022). Is it worth the risk? Clinical and radiographic outcomes 24 months after reverse shoulder arthroplasty in an advanced geriatric population. JSES Int.

[15] Foruria A.M., Sperling J.W., Ankem H.K., Oh L.S., Cofield R.H. (2010). Total shoulder replacement for osteoarthritis in patients 80 years of age and older. J Bone Joint Surg Br.

[16] Heifner J.J., Kumar A.D., Wagner E.R. (2021). Glenohumeral osteoarthritis with intact rotator cuff treated with reverse shoulder arthroplasty: a systematic review. J Shoulder Elbow Surg.

[17] Holschen M., Korting M., Khourdaji P., Bockmann B., Schulte T.L., Witt K.A. (2022). Treatment of proximal humerus fractures using reverse shoulder arthroplasty: do the inclination of the humeral component and the lateral offset of the glenosphere influence the clinical outcome and tuberosity healing?. Arch Orthop Trauma Surg.

[18] Iriberri I., Candrian C., Freehill M.T., Raiss P., Boileau P., Walch G. (2015). Anatomic shoulder replacement for primary osteoarthritis in patients over 80 years: outcome is as good as in younger patients. Acta Orthop.

[19] Jensen A.R., Tangtiphaiboontana J., Marigi E., Mallett K.E., Sperling J.W., Sanchez-Sotelo J. (2021). Anatomic total shoulder arthroplasty for primary glenohumeral osteoarthritis is associated with excellent outcomes and low revision rates in the elderly. J Shoulder Elbow Surg.

[20] Jonsson E.O., Ekholm C., Salomonsson B., Demir Y., Olerud P., Collaborators in the SSG (2021). Reverse total shoulder arthroplasty provides better shoulder function than hemiarthroplasty for displaced 3- and 4-part proximal humeral fractures in patients aged 70 years or older: a multicenter randomized controlled trial. J Shoulder Elbow Surg.

[21] Kennedy J., Klifto C.S., Ledbetter L., Bullock G.S. (2021). Reverse total shoulder arthroplasty clinical and patient-reported outcomes and complications stratified by preoperative diagnosis: a systematic review. J Shoulder Elbow Surg.

[22] Kim H., Kim C.H., Kim M., Lee W., Jeon I.H., Lee K.W. (2022). Is reverse total shoulder arthroplasty (rTSA) more advantageous than anatomic TSA (aTSA) for osteoarthritis with intact cuff tendon? A systematic review and meta-analysis. J Orthop Traumatol.

[23] Kriechling P., Hodel S., Paszicsnyek A., Schwihla I., Borbas P., Wieser K. (2022). Incidence, radiographic predictors, and clinical outcome of acromial stress reaction and acromial fractures in reverse total shoulder arthroplasty. J Shoulder Elbow Surg.

[24] Li X., Knutson Z., Choi D., Lobatto D., Lipman J., Craig E.V. (2013). Effects of glenosphere positioning on impingement-free internal and external rotation after reverse total shoulder arthroplasty. J Shoulder Elbow Surg.

[25] Lopiz Y., Garcia-Coiradas J., Serrano-Mateo L., Garcia-Fernandez C., Marco F. (2016). Reverse shoulder arthroplasty for acute proximal humeral fractures in the geriatric patient: results, health-related quality of life and complication rates. Int Orthop.

[26] Mangano T., Cerruti P., Repetto I., Felli L., Ivaldo N., Giovale M. (2016). Reverse shoulder arthroplasty in older patients: is it worth it? A subjective functional outcome and quality of life survey. Aging Clin Exp Res.

[27] Matthews C.J., Wright T.W., Farmer K.W., Struk A.M., Vasilopoulos T., King J.J. (2019). Outcomes of primary reverse total shoulder arthroplasty in patients younger than 65 years old. J Hand Surg Am.

[28] Merolla G., De Cupis M., Walch G., De Cupis V., Fabbri E., Franceschi F. (2020). Pre-operative factors affecting the indications for anatomical and reverse total shoulder arthroplasty in primary osteoarthritis and outcome comparison in patients aged seventy years and older. Int Orthop.

[29] Mowbray J., Van Niekerk M., Frampton C., Hirner M. (2022). The outcomes of shoulder arthroplasty in those aged >/=70 years with glenohumeral arthritis: a New Zealand Joint Registry study. J Shoulder Elbow Surg.

[30] Murphy S.L., Kochanek K.D., Xu J., Arias E. (2021). Mortality in the United States, 2020. NCHS Data Brief.

[31] Padegimas E.M., Maltenfort M., Lazarus M.D., Ramsey M.L., Williams G.R., Namdari S. (2015). Future patient demand for shoulder arthroplasty by younger patients: national projections. Clin Orthop Relat Res.

[32] Poondla R.K., Sheth M.M., Heldt B.L., Laughlin M.S., Morris B.J., Elkousy H.A. (2021). Anatomic and reverse shoulder arthroplasty in patients 70 years of age and older: a comparison cohort at early to midterm follow-up. J Shoulder Elbow Surg.

[33] Puzzitiello R.N., Nwachukwu B.U., Agarwalla A., Cvetanovich G.L., Chahla J., Romeo A.A. (2020). Patient satisfaction after total shoulder arthroplasty. Orthopedics.

[34] Sasanuma H., Iijima Y., Saito T., Kanaya Y., Yano Y., Fukushima T. (2020). Clinical results of reverse shoulder arthroplasty for comminuted proximal humerus fractures in elderly patients: a comparison between nonporous stems versus trabecular metal stems. JSES Int.

[35] Sebastia-Forcada E., Cebrian-Gomez R., Lizaur-Utrilla A., Gil-Guillen V. (2014). Reverse shoulder arthroplasty versus hemiarthroplasty for acute proximal humeral fractures. A blinded, randomized, controlled, prospective study. J Shoulder Elbow Surg.

[36] Shah S.S., Fu M.C., Ling D., Wong A., Warren R.F., Dines D.M. (2021). The comparative effect of age on clinical outcomes following anatomic total shoulder arthroplasty and reverse total shoulder arthroplasty. Orthopedics.

[37] Shah S.S., Roche A.M., Sullivan S.W., Gaal B.T., Dalton S., Sharma A. (2021). The modern reverse shoulder arthroplasty and an updated systematic review for each complication: part II. JSES Int.

[38] Shimada Y., Takahashi N., Sugaya H., Matsuki K., Tokai M., Hashimoto E. (2021). Clinical outcomes of anatomic total shoulder arthroplasty for primary shoulder osteoarthritis did not differ between elderly and younger Japanese patients. JSES Rev Rep Tech.

[39] Simon M.J.K., Coghlan J.A., Bell S.N. (2022). Shoulder replacement in the elderly with anatomic versus reverse total prosthesis? A prospective 2-year follow-up study. J Clin Med.

[40] Singh J.A., Ramachandran R. (2015). Age-related differences in the use of total shoulder arthroplasty over time: use and outcomes. Bone Joint J.

[41] Slim K., Nini E., Forestier D., Kwiatkowski F., Panis Y., Chipponi J. (2003). Methodological index for non-randomized studies (minors): development and validation of a new instrument. ANZ J Surg.

[42] Testa E.J., Yang D., Steflik M.J., Owens B.D., Parada S.A., Daniels A.H. (2022). Reverse total shoulder arthroplasty in patients 80 years and older: a national database analysis of complications and mortality. J Shoulder Elbow Surg.

[43] Teusink M.J., Otto R.J., Cottrell B.J., Frankle M.A. (2014). What is the effect of postoperative scapular fracture on outcomes of reverse shoulder arthroplasty?. J Shoulder Elbow Surg.

[44] Triplet J.J., Everding N.G., Levy J.C., Formaini N.T., O'Donnell K.P., Moor M.A. (2015). Anatomic and reverse total shoulder arthroplasty in patients older than 80 years. Orthopedics.

[45] Wright T.W., Flurin P.H., Crosby L., Struk A.M., Zuckerman J.D. (2015). Total shoulder arthroplasty outcome for treatment of osteoarthritis: a multicenter study using a contemporary implant. Am J Orthop (Belle Mead NJ).

[46] Wright M.A., Keener J.D., Chamberlain A.M. (2020). Comparison of clinical outcomes after anatomic total shoulder arthroplasty and reverse shoulder arthroplasty in patients 70 years and older with glenohumeral osteoarthritis and an intact rotator cuff. J Am Acad Orthop Surg.

[47] Young A.A., Walch G., Pape G., Gohlke F., Favard L. (2012). Secondary rotator cuff dysfunction following total shoulder arthroplasty for primary glenohumeral osteoarthritis: results of a multicenter study with more than five years of follow-up. J Bone Joint Surg Am.

[48] Zumstein M.A., Pinedo M., Old J., Boileau P. (2011). Problems, complications, reoperations, and revisions in reverse total shoulder arthroplasty: a systematic review. J Shoulder Elbow Surg.

